# Diaphragmatic Endometriosis—A Single-Center Retrospective Analysis of the Patients’ Demographics, Symptomatology, and Long-Term Treatment Outcomes

**DOI:** 10.3390/jcm12206455

**Published:** 2023-10-11

**Authors:** Antoine Naem, Argyrios Andrikos, Alin Stefan Constantin, Michael Khamou, Dimitrios Andrikos, Antonio Simone Laganà, Rudy Leon De Wilde, Harald Krentel

**Affiliations:** 1 Department of Obstetrics, Gynecology, Gynecologic Oncology and Senology, Bethesda Hospital Duisburg, 47053 Duisburg, Germany; argiandrikos@yahoo.gr (A.A.); di.andrikos@gmail.com (D.A.); krentel@cegpa.org (H.K.); 2 Faculty of Mathematics and Computer Science, University of Bremen, 28359 Bremen, Germany; 3 Department of Obstetrics and Gynecology, Albertinen Hospital, 22457 Hamburg, Germany; constantinalin@gmail.com; 4 Department of Radiology, Bethesda Hospital Duisburg, 47053 Duisburg, Germany; 5 Unit of Obstetrics and Gynecology, “Paolo Giaccone” Hospital, Department of Health Promotion, Mother and Child Care, Internal Medicine and Medical Specialties (PROMISE), University of Palermo, 90127 Palermo, Italy; antoniosimone.lagana@unipa.it; 6 Clinic of Gynecology, Obstetrics and Gynecological Oncology, University Hospital for Gynecology, Pius-Hospital Oldenburg, Medical Campus University of Oldenburg, 26121 Oldenburg, Germany

**Keywords:** endometriosis, diaphragm, laparoscopy, ablation, excision, shoulder pain, recurrence

## Abstract

Diaphragmatic endometriosis is rare and forms 0.67–4.7% of all endometriosis cases. Evidence regarding its optimal management is lacking. In this study, we retrospectively analyzed the patient characteristics and long-term treatment outcomes of diaphragmatic endometriosis patients. Over a 4-year period, 23 patients were diagnosed with diaphragmatic endometriosis. The majority of patients had coexisting deep pelvic endometriosis. Cyclic upper abdominal pain was reported by 60.9% of patients, while cyclic chest and shoulder pain were reported by 43.5% and 34.8% of patients, respectively. Most patients were treated with laparoscopic lesion ablation, while 21.1% were treated with minimally invasive excision. The mean follow-up time was 23.7 months. Long-lasting resolution of the chest, abdominal, and shoulder pain occurred in 50%, 35.7%, and 25% of patients, respectively. Nonetheless, 78.9% of patients reported major improvement in their symptoms postoperatively. Significantly higher rates of postoperative shoulder, abdominal, and chest pain were observed in patients who received postoperative hormonal therapy compared with those who did not. All patients treated expectantly remained stable. Therefore, we recommend treating diaphragmatic endometriosis only in symptomatic patients. The risk of incomplete surgery should be minimized by a multidisciplinary diagnostic and therapeutic approach with a careful assessment of the diaphragm and the thoracic cavity.

## 1. Introduction

Endometriosis is a chronic inflammatory estrogen-dependent disease that affects 10% of women of reproductive age [1]. Endometriosis is defined by the presence of endometrial-like glands and stroma out of the uterus. Stromal endometriosis, on the other hand, is a special phenotype of endometriosis where the endometriotic stroma exists without the glandular component of endometriosis [2]. Although this subtype is rarely mentioned, it was reported in almost 45% of biopsies with pelvic endometriosis [3].

Endometriosis is a debilitating disease that interferes negatively with the patients’ daily activities and subsequently can cause a decreased quality of life [4]. This is mainly attributed to the severity of the endometriosis-associated pain. The most commonly reported symptoms of endometriosis are chronic pelvic pain, dysmenorrhea, and dyspareunia [5]. Moreover, it is reported that 20–50% of endometriosis patients are affected by infertility [6]. Despite the relatively high prevalence of endometriosis and its grave consequences, it remains an under-recognized disease, with a delay of 7 years between the symptoms’ onset and diagnosis [7].

Endometriosis is known to be predominantly an intrapelvic disease, with the ovaries, pouch of Douglas, and uterosacral ligaments being its most common locations [8,9]. However, extrapelvic endometriosis coexists in almost 9% of patients with a concomitant pelvic disease [10]. Thoracic and diaphragmatic endometriosis are two of the most commonly described localizations of extrapelvic endometriosis [11]. Diaphragmatic endometriosis was first described by Brews in 1954 [12]. Its estimated prevalence ranges between 0.67% and 4.7% [13,14].

The exact pathogenesis mechanisms of endometriosis have not been precisely determined yet. The retrograde menstruation and embryonic cell rest theories are largely adopted and debated. Nonetheless, both theories have their own pros and cons, and none of them can describe all localizations and manifestations of endometriosis [15]. Similarly, thoracic and diaphragmatic endometriosis have been explained by the migration of either endometrial or endometriotic cells from the pelvis to the upper abdomen with the circulating peritoneal fluid [16]. Others believe that diaphragmatic and thoracic endometriosis originates from the metaplastic transformation of the peritoneal and pleural epithelium, since the pleura, peritoneum, and endometrium share a coelomic origin [17,18]. It should be noted that thoracic endometriosis refers to the presence of endometriotic implants within the thoracic cavity, i.e., on the diaphragmatic and parietal pleura and the visceral pleura as well [19], while diaphragmatic endometriosis when used solely often refers to endometriotic implants encountered on the visceral (abdominal) side of the diaphragm [20]. Although these appear to be two different entities, a strong association and a potentially causative relationship between diaphragmatic and thoracic endometriosis have been described in the literature [21], with the former being the source of the latter [13,22].

Even though diaphragmatic endometriosis could cause symptoms like cyclic or noncyclic shoulder, arm, chest, or upper quadrant abdominal pain [20,22], it is most frequently diagnosed incidentally during a laparoscopic surgery for a coexisting pelvic endometriosis [23]. This could be due to the lack of correlation between pain symptoms in the upper side of the body and endometriosis by the patients and examining physicians, which consequently yields in a diagnostic delay of about 2 years from the start of symptoms [24].

The evidence concerning the optimal management of diaphragmatic endometriosis is lacking due to its relatively low prevalence and the limited awareness of this condition. The published research on both thoracic and diaphragmatic endometriosis so far is limited to few case series, mostly with less than 50 patients and inappropriate follow-up strategy or duration [25]. Additionally, it is thought that this field is prone to publication bias, since most authors are prone to promote positive results only [23]. In this paper, we provide a thorough and detailed description of the characteristics, symptomatology, and long-term treatment outcomes of patients with diaphragmatic endometriosis. We tried to track on a symptom-by-symptom basis the postoperative pain occurrence and recurrence. To our knowledge, this study is considered one of the largest series on diaphragmatic endometriosis, with 23 patients included and followed-up overall.

## 2. Materials and Methods

This is a retrospective observational cohort study that included all patients with a surgical diagnosis of diaphragmatic endometriosis who were admitted to the department of obstetrics and gynecology of Bethesda Hospital Duisburg (Duisburg, Germany) between 1 January 2019 and 31 December 2022. This study was conducted in compliance with the ethical standards of the Declaration of Helsinki (1964) and the guidelines of the Committee on Publication Ethics (COPE) and reported in accordance with the Reporting of studies Conducted using Observational Routinely collected health Data (RECORD) statement [26], made available through the Enhancing the Quality and Transparency of Health Research (EQUATOR) network (www.equator-network.org, accessed on 26 August 2023).

Written informed consent was obtained from each patient regarding the performed interventions and the use of their medical data for research purposes. An institutional review board approval was deemed unnecessary for the conduct of this study according to the internal regulations of our institution, since this research is based on retrospectively collected anonymized medical data and all patients gave their consent individually to participate in this study.

The main inclusion criterion was having a surgical diagnosis of diaphragmatic endometriosis made through the direct inspection of the diaphragm intraoperatively regardless of the presence of a histopathological confirmation of endometriosis. Our decision was made in light of the recent ESHRE guidelines stating that a negative histology does not completely rule out endometriosis [27], while also taking into consideration the technical challenges imposed by the limited operative space in the subphrenic and retrohepatic spaces, which make it difficult to obtain a biopsy with sufficient histology to rule endometriosis in or out, as reported previously [24]. These cases were identified by searching for patients’ records with a documented classification of “*#Enzian F(diaphragm)*” according to the #Enzian classification [28]. Owing to the scarcity of diaphragmatic endometriosis cases, we limited our exclusion criteria to patients unwilling to give informed consent for participation in the study or those who revoked their consent at any time during the study or follow-up periods. Pelvic endometriosis was diagnosed with the guidance of preoperative Transvaginal Ultrasonography (TVUS) scan and/or Magnetic Resonance Imaging (MRI) with a subsequent histopathological confirmation in all patients. The TVUS scans and MRI were performed by a gynecologist specialized in endometriosis imaging and a trained radiologist experienced in the radiologic diagnosis of endometriosis, respectively. All patients were operated under general anesthesia and positioned in the dorsal lithotomy position with standard trocars placement. A close inspection of the diaphragm was performed routinely in every patient with pelvic endometriosis at the beginning of the laparoscopy. When diaphragmatic lesions were observed and a resection was planned, the patients were positioned in the steep reverse Trendelenburg position, and—based on the surgeon’s preference—additional trocars were inserted inferior to the subcostal margin. A 30-degree laparoscope was used in all cases. We did not follow a standard approach to treat diaphragmatic lesions, as the optimal management is still debated. The decision of whether to leave the lesions in situ, ablate, or excise them was conducted on a patient-by-patient basis and based on the pre- and intraoperative findings. Superficial diaphragmatic lesions were either ablated with bipolar electrocoagulation or excised through diaphragmatic peritoneal stripping, as previously described [29]. Full-thickness resection of the diaphragm and subsequent opening of the pleural cavity was performed with monopolar energy or ultrasound dissection. In this situation, the diaphragmatic defects were closed with a continuous reabsorbable suture. Diaphragmatic endometriosis was left in situ in asymptomatic patients.

Data regarding the patients’ demographics, menstrual cycle characteristics, medical and obstetric history, radiological findings, complete #Enzian classification, intraoperative findings, and surgical management were retrospectively collected. The patients were followed-up by a questionnaire-guided telephone interview. Data regarding the hormonal or medical therapies along with their success rates before the index surgery are unavailable. All patients were asked about the presence of pre- and postoperative upper abdominal, shoulder, and chest pain, whether they were reoperated for diaphragmatic endometriosis-related symptoms, received postoperative hormonal treatment regardless of its type and route of administration, and to rate their postoperative condition based on the Patient’s Global Impression of Improvement (PGI-I) scale. The PGI-I scale is a 7-point scale that allows patients to evaluate the extent of improvement or deterioration in their condition compared with their initial state at the start of the therapy. The PGI-I scales are broadly applied in psychiatry, and their utility extends to various other medical and gynecological domains as well. Although the PGI-I scale was validated for urinary incontinence and urogenital prolapse [30,31], its use for the postoperative outcomes of endometriosis surgery has not been validated yet. When a history of infertility was present, the patients were asked about their postoperative wish to conceive and pregnancy occurrence. Where appropriate, the patients were asked about the means of conception and birth outcomes.

All the data were collected from a questionnaire that was specifically designed for this study. The data analysis was performed using descriptive statistics. Continuous variables were expressed as means ± standard deviations or medians with the interquartile ranges (IQR), based on their distribution. Shapiro–Wilk test was used to determine whether or not the data were normally distributed. Categorical variables were expressed as frequencies and valid percentages. Continuous variables were compared by T-Test or Mann–Whitney U-test based on their distribution. Categorical Variables were compared by Chi-Square Test or Fisher’s Exact Test as appropriate. The significance level was set at *p* < 0.050. The statistical analysis was performed using the Statistical Package for Social Sciences (SPSS) software, version 25.0 (SPSS, Chicago, IL, USA).

## 3. Results

### 3.1. General Characteristics

A total of 1237 patients with histologically proven pelvic endometriosis underwent surgery in our department between the years 2019 and 2022. Among them, only 23 patients had coexisting diaphragmatic endometriosis, making its prevalence in our cohort 1.86%. The mean age at presentation was 33.6 ± 5.8 years, and the mean age at menarche was 12.7 ± 1.5 years. The median menstrual cycle length was 28 days (IQR = 1.25), and the menstruation lasted for a mean duration of 5.6 ± 1.6 days. A total of 11 patients (52.4%) reported infertility at the time of presentation. Subsequently, 70.6% of patients were nulligravidae, and 82.4% of them were nulliparous. More than half of the patients (60.4%) had at least one previous surgical intervention for the treatment of pelvic endometriosis. However, none of them received previous diaphragmatic or thoracic surgery. The general characteristics of patients are summarized in Table 1.

### 3.2. Symptomatology

The most commonly reported symptom was dysmenorrhea, as it was reported by 68.2% of patients, followed by cyclic upper abdominal pain (n = 14/23, 60.9%) and cyclic chest pain (n = 10/23, 43.5%). Cyclic right shoulder pain was reported by only 8 patients (34.8%). On the other hand, dyspareunia and dyschezia were reported by 31.8% and 27.3% of patients, respectively. Cyclic apnea and coughing were reported by 4 patients (17.4%). Catamenial or noncatamenial pneumothorax was not reported by any of the included patients.

### 3.3. Pelvic Endometriosis

Deep infiltrating endometriosis in the pelvis was present in 20 out of 23 patients (87%), while three patients had only superficial peritoneal endometriosis. Ovarian endometriomas (#Enzian O) were present in 7 patients (30.4%). Deep endometriosis of the rectovaginal septum (#Enzian A) and parametrium (#Enzian B) was confirmed in 11 (47.8%) and 15 (65.2%) patients, respectively. Rectal endometriosis (#Enzian C) was present in 4 patients (17.4%). Adenomyosis (#Enzian FA) was diagnosed in 12 patients (52.2%). A total of 14 patients (60.9%) were classified with severe endometriosis (Stages III–IV) according to the revised classification of the American Society of Reproductive Medicine (r-ASRM) [32]. All patients had simultaneous excisional surgery for peritoneal and deep pelvic endometriosis in the parametrium and the recto-vaginal septum. In addition, segmental rectal resection was performed in three patients and rectal shaving was performed in one patient with concomitant rectal endometriosis. A summary of the intraoperative findings is presented in Table 2.

### 3.4. Diaphragmatic Endometriosis

**Figure 1:**
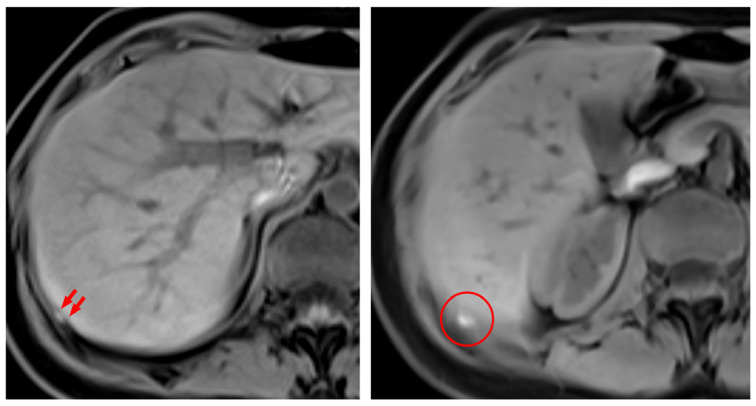
Diaphragmatic endometriosis in a 28-year-old woman. Axial fat-saturated three-dimensional T1-weighted MRI shows high signal intensity nodules attached to the posterior part of the right hemidiaphragm. The red arrows (on the left) point to the endometriotic nodule and the red circle (on the right) demonstrates the location of the endometriotic nodule with the lesion being in the middle.

**Figure 2:**
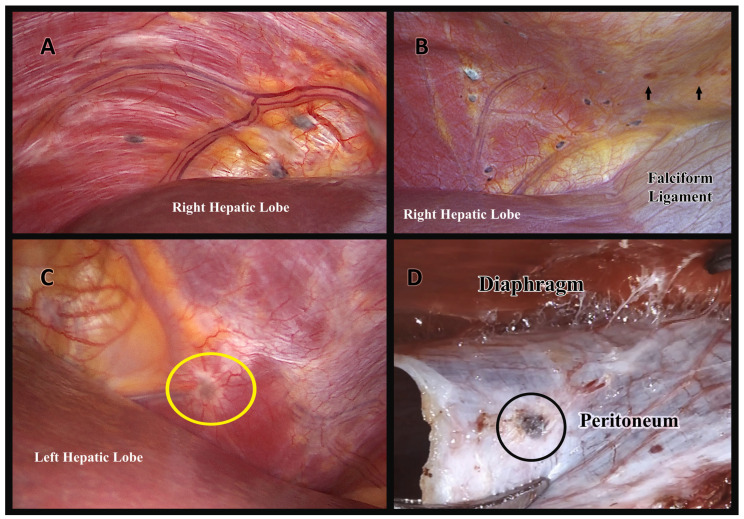
The intraoperative appearances of diaphragmatic endometriosis: (**A**) Puckered black lesions on the right diaphragmatic dome. (**B**) Diaphragmatic endometriosis on the right diaphragmatic dome with yellow–brown “Café au Lait” hemosiderin patches (arrows). (**C**) Left diaphragmatic endometriosis (in the centre of the yellow circle). Note the newly formed blood vessels in the periphery of the lesion, highlighting the angiogenic characteristic of endometriosis. (**D**) Laparoscopic view of the peritoneal stripping technique demonstrating the superficial localization of the diaphragmatic implant (in the centre of the black circle).

**Figure 3:**
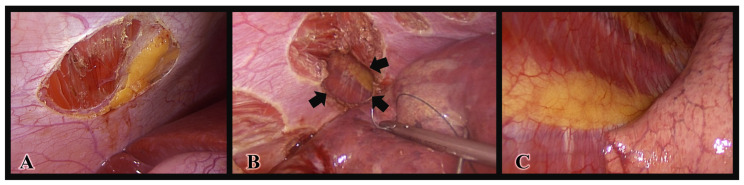
Intraoperative pictures demonstrating the diaphragm’s appearance by the end of the peritoneal stripping (**A**) and full-thickness resection (**B**). The arrows in image B point to the borders of the full thickness resection area. An entry to the thoracic cavity and a subsequent inspection of the parietal and visceral pleura are possible through the defect of the diaphragm after the full-thickness resection (**C**).

A preoperative radiologic diagnosis of diaphragmatic endometriosis by MRI was established in only two patients (Figure 1). Endometriotic implants of the diaphragm were localized on the right hemidiaphragm in 13 patients (68.4%), on the left hemidiaphragm in 2 patients (10.5%), and located on both domes of the diaphragm in 4 patients (21.1%). Some of the lesions were superficially limited to the peritoneum, and others presented as deep nodules with partial or full-thickness infiltration. Figure 2 demonstrates some of the intraoperative appearances of diaphragmatic implants. It is noteworthy that one of the patients with left diaphragmatic endometriosis had endometriotic implants over the serosal surface of the left hepatic lobe. Moreover, one patient had Fitz-Hugh–Curtis syndrome-like adhesions, and another patient had spontaneous minor dehiscence of the diaphragm. The diaphragmatic lesions were excised in the means of ablation, peritoneal stripping, or excision in 19 patients (82.6%), while 4 patients (17.4%) had their lesions left in situ. For those who were operated for diaphragmatic endometriosis, 12 patients (63.2%) were treated with bipolar ablation of all visible lesions, 4 patients (21.1%) were treated with complete surgical excision, and 3 patients (15.8%) were treated with a combination of both approaches (Figure 3). When needed, mobilization of the right liver lobe was performed (n = 3/19, 15.8%). After full-thickness resection of the diaphragm with subsequent entry to the thoracic cavity, suturing of the diaphragm was performed with continuous reabsorbable suture. A thoracic drain was unnecessary. No intra- or postoperative complications related to diaphragmatic surgery occurred in any patient. Postoperative complications were related to a concomitant deep endometriosis excisional surgery performed in the pelvis (Table 3). It is noteworthy that the complication rate, patient satisfaction, and recurrence rates were comparable between patients who underwent endometriosis excision and ablation.

### 3.5. Patient Follow-Up

All patients were followed-up postoperatively. The mean follow-up time was 23.7 ± 13 months and ranged between 6 and 49 months. The follow-up questionnaire was structured on a symptom-by-symptom basis. A total of eight patients reported preoperative cyclic shoulder pain. At the 6th and 12th postoperative months, 62.5% (n = 5/8) and 50% (n = 4/8) of patients were pain-free. After one year postoperatively, shoulder pain recurred in six out of the eight patients who had preoperative shoulder pain (75%), i.e., complete symptom relief occurred in two patients only (25%). On the other hand, two patients that were treated with ablation developed de novo postoperative shoulder pain.

Cyclic upper abdominal pain was documented in 14 patients preoperatively. Out of these, only five patients (35.7%) had their upper abdominal pain completely relieved at the time of follow-up. Similarly, half of the patients (n = 5/10, 50%) who reported preoperative cyclic chest pain had their symptoms completely treated when approached for follow-up. It is noteworthy that all four patients who had their lesions left in situ either remained asymptomatic (three/four patients) or the pain remained stable without aggravation (1 patient). Table 3 demonstrates the characteristics and follow-up outcomes of all patients who were asymptomatic and treated expectantly. The reoperation rate was very low in our cohort, since only one patient (4.3%) received a second surgery for diaphragmatic endometriosis. Notably, this patient had new lesion formation near the site of the previous ablation. The use of the recommended postoperative hormonal treatment to further control diaphragmatic-endometriosis-related symptoms was low, as it was reported by five patients only (21.7%). The results of the PGI-I scores are in favor of surgical management. After the exclusion of patients who did not receive diaphragmatic surgery, a total of 15 out of 19 patients (78.9%) reported major improvement postoperatively, while only 4 patients (21.1%) reported little to no improvement at all.

Regarding the postoperative fertility status, 9 out of 11 patients tried actively to conceive during the postsurgical period. Four patients managed to achieve a clinical pregnancy postoperatively, making the clinical pregnancy rate 44.4%. Three patients conceived spontaneously and one by means of intracytoplasmic sperm injection (ICSI). At the time of follow-up, two patients had given birth to live babies, and two patients were still pregnant. Table 4 provides a summary of the performed procedures, intraoperative complications, and follow-up results.

### 3.6. Postoperative Hormonal-Treatment-Associated Factors

The patients were divided into two groups based on the postoperative administration of hormonal treatment to detect any variable that may be associated with it. The two groups were comparable in terms of age at presentation, age at menarche, menstrual cycle length, menstruation duration, previous surgical history, gravidity, parity, pelvic-endometriosis-related symptoms, intraoperative findings, applied treatment modalities, intraoperative complication rate, and follow-up duration (*p* > 0.05). However, the infertility rate differed significantly between the two groups. Patients who did not receive postoperative hormonal treatment had significantly higher infertility rates compared with patients who received it (64.7% vs. 0%, *p* = 0.035). Patients who received postoperative hormonal therapy had significantly higher rates of preoperative shoulder pain compared with those who did not (100% vs. 16.7%, *p* = 0.002). There was no significant difference between the two groups in the rates of preoperative upper abdominal pain and chest pain (*p* > 0.05). It is noteworthy that patients who received postoperative hormonal therapy had higher rates of postoperative shoulder pain (100% vs. 16.7%, *p* = 0.002), postoperative upper abdominal pain (100% vs. 33.3%, *p* = 0.014), and postoperative chest pain (60% vs. 11.1%, *p* = 0.048) compared with patients who did not receive hormonal treatment. The outcomes of the PGI-I scale were grouped into “Significant Improvement” when the responses were “Very Much Better” and “Much Better” and “Insignificant Improvement” when the responses were “A little Better” and “No Change”. Remarkably, all patients who did not receive postoperative hormonal treatment (100%) reported a significant improvement, and none of them reported an insignificant improvement (0%). On the other hand, 80% of patients who received postoperative hormonal therapy reported insignificant improvement, and 20% of them reported a significant improvement. These differences are statistically significant (*p* = 0.001). Table 5 demonstrates a comparison between the two groups in terms of the patients’ characteristics, presenting symptoms, surgical management and follow-up outcomes.

## 4. Discussion

Endometriosis is one of the most common gynecologic diseases worldwide. It is estimated to be the second most common gynecologic condition proceeding the uterine fibroids [33]. Yet, much of the endometriosis-related etiologies and manifestations are not studied sufficiently [15]. The optimal management of diaphragmatic endometriosis remains a point of debate due to the relatively low number of diaphragmatic endometriosis cases and the low level of suspicion for this condition. To our knowledge, our study could be considered one of the largest series of diaphragmatic endometriosis, with a total of 23 patients that were operated and followed-up. Despite the reported high satisfaction rates in our study, pain persistence and recurrence were observed regardless of the used surgical approach. Moreover, we demonstrated that fertile patients, patients suffering from persistent or recurring symptoms, and patients who do not notice a significant postoperative improvement have a higher tendency to take hormonal therapies postoperatively.

The prevalence of diaphragmatic endometriosis in our center is 1.86%, which is very close to what was previously reported [29]. Although patients with endometriosis were reported to have abnormal uterine peristalsis with longer and heavier menstrual cycles [34,35], we did not notice abnormal menstrual patterns in our cohort. Only six patients reported menorrhagia; five of them had coexisting adenomyosis. All of our patients had coexisting pelvic endometriosis and around 87% of them had deep infiltrating endometriosis of the parametrium, rectovaginal septum, and/or the rectum. Moreover, most patients were classified with stages III and IV endometriosis according to the r-ASRM classification. Additionally, approximately 50% of our cohort had coexisting adenomyosis proven through transvaginal ultrasonographic and laparoscopic findings. Similarly, Ceccaroni et al. reported in their two studies that the majority of diaphragmatic endometriosis patients had stage IV pelvic endometriosis [29,36]. This association was also emphasized by a recent study demonstrating that most diaphragmatic endometriosis patients (78.4%) have stage III–IV endometriosis [14]. Interestingly, diaphragmatic endometriosis was suggested to be an indirect sign of severe pelvic disease [36].

A strong association between thoracic endometriosis and infertility was previously reported in the literature [37]. Although the same correlation has not been established yet in diaphragmatic endometriosis, patients with diaphragmatic endometriosis were reported to have higher rates of infertility compared with endometriosis patients without diaphragmatic involvement [14]. The infertility rate in our cohort is 52.4%, which is slightly higher than what is reported in the literature. According to Pagano et al. [14], the infertility rate in patients with diaphragmatic endometriosis is estimated to be 49.2%. In another study, the infertility rate was reported to be 39.2% [36]. Nonetheless, Wetzel et al. [24] reported a remarkably higher infertility rate of 66.7%. This in turn suggests a strong correlation between diaphragmatic endometriosis, advanced pelvic endometriosis, and infertility.

In line with what was previously reported by Ceccaroni et al. [36], dysmenorrhea was the most frequently reported symptom in our cohort. On the other hand, cyclic shoulder pain was present in 34.8% of our patients, in comparison with 3.2% in the study by Ceccaroni et al. [36] and 75% in the cohort of Wetzel et al. [24]. The rates of cyclic chest pain in our cohort and Wetzel et al.’s [24] study exceed considerably the rates reported by Ceccaroini et al. [36]. However, the rates of cyclic upper abdominal pain in our study are remarkably higher than those of the two aforementioned studies [24,36]. The underlying reason for these variations in the prevalence of diaphragmatic endometriosis symptoms is hard to determine. A possible explanation could be the decreased detection of some symptoms, since patients may not report pain in the upper body to their gynecologist, and the examining physician may not investigate symptoms related to diaphragmatic endometriosis. According to the results of an international patient survey, upper abdominal pain, chest pain, and shoulder pain are reported by 68%, 64%, and 54% of patients [23]. This may indicate that higher rates of these symptoms are observed when patients are directly asked about them, which adds more justification to our assumption.

Our results contribute further evidence towards the course of diaphragmatic endometriosis. All four patients who were treated expectantly remained stable during the study period. This is partly in line with the study by Nezhat et al. [22], since they reported that one of three patients became symptomatic. Although data regarding the natural history of diaphragmatic endometriosis are lacking, it is worth mentioning that pelvic endometriosis was found to remain stable in 29–50% of cases and regress in 42–50% of cases during follow-up periods of at least 6 months [38,39]. Nonetheless, caution should be exercised when interpreting our results because expectantly treated patients were only followed-up for 6 to 14 months. Although unlikely, this follow-up period might be insufficient to completely exclude the chance of endometriosis progression.

Despite the high satisfaction rates observed in our cohort, the diaphragmatic-endometriosis-related pain recurrence rates are higher than expected. The most significant pain relief occurred in the first 6 and 12 months postoperatively. Afterwards, the pain recurred. It is noteworthy that detailed data on pain recurrence patterns are missing in the published literature. In the study by Ceccaroni et al. [29], the follow-up period was limited to 6 months only. The authors observed no recurrences at that time. In their later study [36], the authors did not mention either the follow-up intervals or follow-up strategy. In the study by Pagano et al. [14], the authors stated that only 6.2% of their patients underwent a second operation for symptom persistence. Their reoperation rate is comparable to ours, but the authors did not mention relevant information regarding the persistence or recurrence of postoperative pain symptoms. In other words, patients may have had pain recurrence or persistence but did not undergo a second operation. Wetzel et al. [24] reported a recurrence rate of 29.2% at a maximum follow-up period of 39 months. The observed difference between our results and those of Wetzel et al. [24] could be attributed either to the longer follow-up period in our study or the differences in the surgical techniques. Notably, the excision of diaphragmatic lesions was more frequently applied in the series of Wetzel et al. [24] and Ceccaroni et al. [29,36] compared with ours. Another potential explanation of the recurrences observed in our cohort is the coexistence of thoracic endometriosis on the parietal, diaphragmatic or visceral pleura. Diaphragmatic endometriosis as visualized by laparoscopy was present in 89% of patients with catamenial pneumothorax [21], which is supposed to be a direct sign of the thoracic endometriosis syndrome. It should be noted that diaphragmatic pleural involvement was noted in 100% of patients with thoracic endometriosis [40]. Wetzel et al. [24] reported better treatment outcomes in patients who received Video-Assisted Thoracoscopy (VATs). Although we cannot recommend VAT to every patient with diaphragmatic lesions, it seems highly recommendable to explore the thoracic cavity in symptomatic cases through VAT by an experienced thoracic surgeon to ensure the complete detection and excision of all lesions involving the thoracic cavity and the abdominal side of the diaphragm. This conclusion is based on our belief that direct inspection of the diaphragm and thoracic cavity is the most precise way available for detecting endometriosis, owing to the limited reliability of the current imaging methods. In fact, it is unclear how precise the available radiologic imaging methods are in diagnosing and detecting all lesions of the diaphragm and thorax, especially in small-sized lesions.

Radiologically, diaphragmatic endometriosis may manifest as high-signal-intensity nodules on fat-suppressed T1-weighted MRI. Diaphragmatic endometriosis was also reported to take the shape of plaques or micronodules in MRI [41]. Another manifestation reported by Querci et al. [42] is the “air-filled bubble” lesions in the coronal planes. Another study reported a sensitivity of 78–83% in MRI in diagnosing diaphragmatic endometriosis [43]. It is noteworthy that those results are drawn from patients who underwent a chest MRI for the suspicion of diaphragmatic endometriosis. In other words, MRI might only be useful when diaphragmatic endometriosis is suspected. On the other hand, its diagnostic performance remains questionable, since no study to date assessed how accurate it is in determining the lesions’ count and distribution, which are of paramount importance for a precise preoperative surgical mapping and planning.

Another important surgical step in order to avoid the persistence of lesions seems to be the mobilization of the liver with subsequent inspection of the posterior part of the diaphragm. Liver mobilization was performed in 15.7% of cases compared with 16.7% in the series of Ceccaroni et al. [36]. In the rest of the published studies, data regarding the performance of liver mobilization and its frequency are lacking [14,24,29]. According to an earlier study by David Redwine, lesions on the anterior part of the diaphragm (or “sentinel lesions” as named by the author) often coexist with lesions on the posterior side of the diaphragm, which tend to be the more serious aspect of diaphragmatic endometriosis [13]. Therefore, it is plausible to think that undiagnosed lesions could be a source for pain persistence and recurrence, but it also remains unclear whether liver mobilization could guarantee better outcomes. It should be noted that liver mobilization does not eliminate the need of VATs to guarantee a complete detection of endometriotic lesions of the diaphragm. In some cases, some lesions may still be invisible despite the mobilization of the liver, as it was demonstrated recently [44].

Although diaphragmatic surgery for endometriosis has been widely reported and described in the literature, accurate follow-up data demonstrating its efficacy are lacking. It is reasonable to think that surgery reduced the pain intensity in our cohort when considering the high satisfaction rates observed. Therefore, it is of utmost importance to appropriately council the patients and clarify explicitly what to expect from the surgery. One possibility to be considered is that surgery may be ineffective in this region and is better to be avoided if the effect size is minimal when compared with the risks of such operations [45].

Another potential explanation for postoperative pain persistence or recurrence is the surgical trauma itself. In our study, two patients reported new onset symptoms after the surgery, which might be related to phrenic nerve irritation, scarring, or pain centralization. However, these remain speculations and lack evidence.

In our study, only 21% of the patients took the recommended hormonal treatment postoperatively. The relatively high percentage of infertility in patients with diaphragmatic endometriosis and the fact that these patients tried to conceive just after the surgery explains the low rate of hormonal therapy acceptance. This postulation is further justified by the significantly higher infertility rate found in the group of patients who did not receive postoperative hormonal therapy. Moreover, it seems that the presence of preoperative shoulder pain and its postoperative persistence are factors associated with the acceptance of hormonal therapy among patients with diaphragmatic endometriosis. The same applies to postoperative upper abdominal and chest pain. Furthermore, it seems that patients tend to accept postoperative hormonal therapy when they are unsatisfied with the operation results, as reflected by the responses to the PGI-I questionnaire. Most patients who did not receive hormonal therapy reported significant improvement in comparison with 20% of those who received postoperative hormonal treatment. To the best of our knowledge, these results were not previously reported in the literature.

The optimal treatment for diaphragmatic endometriosis is controversial and hard to decide upon in light of current evidence. However, asymptomatic diaphragmatic endometriosis should be left in situ. When diaphragmatic-endometriosis-related symptoms are reported by patients, surgical excision of the diaphragmatic lesions in the mean of peritoneal stripping or ablation through argon beam coagulation, diathermocoagulation, bipolar energy, or plasma energy seem equally effective. This is mainly attributed to the predominance of superficial diaphragmatic lesions. Ceccaroni et al. [36] reported that only 27% of cases had deep infiltration of the diaphragm, and full-thickness excision was required in only 16.7% of the included population. However, whenever full-thickness diaphragmatic lesions are encountered, a full-thickness resection of the diaphragm with subsequent opening of the thoracic cavity seems unavoidable. In our opinion, such interventions are better performed with a multidisciplinary team with a thoracic surgeon involved.

Our paper has many strengths and limitations. The relatively large sample size and long postoperative follow-up duration in addition to the high compliance with follow-up (100% of patients) are the main strengths. In addition, we provided a detailed description of the postoperative course of the majority of symptoms related to diaphragmatic endometriosis. Moreover, our study is the first to measure the patient’s satisfaction by the PGI-I scale. The study’s main limitations are inherent in the retrospective nature of the design. Some Important variables like the operation duration, count, and shape of diaphragmatic lesions are missing. In addition, patients were asked about their preoperative symptoms at the time of presentation and during the follow-up interview. This may be subject to recall bias and also could yield in hindsight bias. However, the symptoms reported upon follow-up matched the records in all but three patients.

## 5. Conclusions

Investigating the symptoms related to diaphragmatic endometriosis should be routinely carried out during the examination of every endometriosis patient. Asymptomatic lesions or those incidentally diagnosed should be left in situ. In symptomatic patients, presurgical assessment should include thoracic and abdominal MRI and a multidisciplinary approach including thoracic surgeons when needed. Laparoscopic examination of the anterior and posterior diaphragm is recommended to ensure full lesion detection. The performance of liver mobilization and VATs in every symptomatic patient should be analyzed in larger multicentric trials. However, the radicality of the surgery should be tailored to the patient’s symptoms and the expected benefits and risks of the operation. Postoperative hormonal therapy is apparently accepted by patients when their symptoms persist or recur postoperatively and when they are unsatisfied with the improvement extent of their symptoms postoperatively.

## Figures and Tables

**Table 1:** General characteristics and symptoms at the time of presentation of the included patients. Results are presented as medians ± standard deviation, medians with interquartile range, or frequencies and valid percentages, as appropriate. IQR: Interquartile Range.

Characteristics	Diaphragmatic Endometriosis Cases (n= 23)
Age (years)	33.6 ± 5.8
Menarche Age (years)	12.7 ± 1.5
Menstrual Cycle Length (days)	28 (IQR = 1.25)
Menstruation Duration (days)	5.6 ± 1.6
Previous Surgical History (%)	13 (68.4%)
Gravidity	
Nulligravidae (%)	12 (70.6%)
Primigravida (%)	5 (29.4%)
Parity	
Nulliparous (%)	14 (82.4%)
Primiparous (%)	3 (17.6%)
Infertility (%)	11 (52.4%)
Symptoms	
Dysmenorrhea (%)	15 (68.2%)
Menorrhagia (%)	6 (27.3%)
Dyspareunia (%)	7 (31.8%)
Dyschezia (%)	6 (27.3%)
Hematochezia (%)	2 (9.1%)
Constipation (%)	2 (9.1%)
Dysuria (%)	5 (21.7%)
Cyclic Shoulder pain (%)	8 (34.8%)
Cyclic Upper Abdominal Pain (%)	14 (60.9%)
Cyclic Chest Pain (%)	10 (43.5%)

**Table 2:** The intraoperative findings classified according to the #Enzian classification and the revised classification of the American Association of Reproductive Medicine (r-ASRM).

Classification	Diaphragmatic Endometriosis Cases n (%)
#ENZIAN P	23 (100%)
#ENZIAN O (Left)	6 (26.1%)
#ENZIAN O (Right)	4 (17.4%)
#ENZIAN T (Left)	6 (26.1%)
#ENZIAN T (Right)	4 (17.4%)
Deep Endometriosis	20 (87%)
#ENZIAN A	11 (47.8%)
#ENZIAN B (Left)	15 (65.2%)
#ENZIAN B (Right)	14 (60.9%)
#ENZIAN C (Rectum)	4 (17.4%)
#ENZIAN FA	12 (52.2%)
#ENZIAN F(Diaphragm)	23 (100%)
Right-Sided	13 (68.4%)
Left-Sided	2 (10.5)
Bilateral	4 (21.5%)
r-ASRM Stage I	2 (8.7%)
r-ASRM Stage II	7 (30.4%)
r-ASRM Stage III	8 (34.8%)
r-ASRM Stage VI	6 (26.1%)

**Table 3:** Symptoms and related surgical findings of patients who were not treated for diaphragmatic endometriosis.

Case	Age (Years)	Presenting Complains	#ENZIAN	r-ASRM Stage	Follow-Up Duration (Months)	Follow-Up Outcomes
#1	34	Dysmenorrhea Menorrhagia Dyspareunia Infertility	P2, O0/0, T1/1, A0, B1/1, C0, FA, F(Diaphragm)	III	11	No New Diaphragmatic-Endometriosis-Related Symptoms Occurred. No Postoperative Hormonal Treatment or Reoperation Were Reported.
#2	34	Dysmenorrhea Dyspareunia Infertility	P3, O1/2, T1/1, A2, B2/2, C2, FI (Terminal Ileum), F(Diaphragm)	IV	14	
#3	25	Dysmenorrhea Menorrhagia	P3, O2/2, T1/1, A0, B2/2, C0, FI (Cecum + Appendix), F(Diaphragm)	IV	8	
#4	30	Dysmenorrhea Menorrhagia	P3, O0/0, T0/0, A1, B2/2, C0, FA, FI(Cecum + Appendix), F(Diaphragm)	III	6	

Please note that patient #1 had stable pre- and postoperative upper abdominal pain, while the other three patients were asymptomatic pre- and postoperatively. r-ASRM: Revised Classification of the American Society of Reproductive Medicine.

**Table 4:** The surgical management of diaphragmatic endometriosis and the follow-up results. Values are presented as means ± standard deviation or frequencies and valid percentages, as appropriate.

Diaphragmatic Endometriosis Cases (n = 23)	
Treatment of Diaphragmatic Endometriosis	
Bipolar Coagulation (%)	12 (52.2%)
Excision (%)	4 (17.4%)
Excision and Coagulation (%)	3 (13%)
Left In Situ (%)	4 (17.4%)
Postoperative Complications	
Overall Complications Rate (%)	6 (26.1%)
Clavien–Dindo Class 1 (%) Minimal Pneumothorax	2 (33.3%)
Clavien–Dindo Class 2 (%) Urinary Tract Infection Vaginal Suture Infection	2 (33.3%)
Clavien–Dindo Class 3b (%) Pouch of Douglas Hematoma Left ureteral stenosis	2 (33.3%)
Follow-up Results	
Follow-up Duration (Months)	23.7 ±13
Cyclic Shoulder pain (n = 8)	6 (75%)
Cyclic Upper Abdominal Pain (n = 14)	9 (64.3%)
Cyclic Chest Pain (n = 10)	5 (50%)
Reoperation (n = 23)	1 (4.3%)
Postoperative Hormonal Therapy (%)	5 (21.7%)
Clinical Pregnancy Rate (n = 9)	4 (44%)
Live Birth Rate (n = 4)	2 (50%)
Ongoing Pregnancy Rate (n = 4)	2 (50%)
Patient Global Impression of Improvement	
Very Much Better (%)	10 (43.5%)
Much Better (%)	9 (39.1%)
A Little Better (%)	2 (8.7%)
No Change at All (%)	2 (8.7%)
Minimally Worse (%)	0 (0%)
Much Worse (%)	0 (0%)
Very Much Worse (%)	0 (0%)

**Table 5:** A Comparison between patients who received and did not receive postoperative hormonal therapy.

Characteristics	Treatment Group (n = 5)	Control Group (n = 18)	*p*-Value
Age (years)	34.4 ± 5.8	33.3 ± 5.9	0.72
Menarche Age (years)	13 (IQR = 3)	13 (IQR = 2.25)	0.63
Menstrual Cycle Length (days)	28 (IQR = 3.5)	28 (IQR = 2)	0.64
Menstruation Duration (days)	6.5 (IQR = 1.75)	5 (IQR = 2)	0.17
Gravidity (%)	1 (20%)	4 (33.3%)	1
Parity (%)	1 (20%)	2 (16.7%)	0.4
Abortions (%)	1 (20%)	2 (16.7%)	0.4
Previous Surgical History (%)	4 (80%)	9 (60%)	0.25
Infertility (%)	0 (0%)	11 (64.7%)	**0.03**
Preoperative Symptoms			
Dysmenorrhea (%)	3 (60%)	12 (70.6%)	1
Menorrhagia (%)	1 (20%)	5 (29.4%)	1
Dyspareunia (%)	0 (0%)	7 (41.2%)	0.14
Dyschezia (%)	0 (0%)	6 (35.3%)	0.27
Hematochezia (%)	0 (0%)	2 (11.8%)	1
Constipation (%)	0 (0%)	2 (11.8%)	1
Dysuria (%)	0 (0%)	5 (27.8%)	1
Cyclic Shoulder pain (%)	5 (100%)	3 (16.7%)	**0.002**
Cyclic Upper Abdominal Pain (%)	5 (100%)	9 (50%)	0.12
Cyclic Chest Pain (%)	4 (80%)	6 (33.3%)	0.13
Surgical Treatment of Diaphragmatic Endometriosis			
Bipolar Coagulation (%)	4 (80%)	8 (57.1%)	0.6
Excision Only (%)	0 (0%)	4 (28.6%)	
Excision and Coagulation (%)	1 (20%)	2 (14.3%)	
Intraoperative Complications (%)	2 (40%)	4 (22.2%)	0.58
Postoperative Outcomes			
Follow-up Duration (Months)	18 (IQR = 27.5)	25.5 (IQR = 20.75)	0.91
Cyclic Shoulder pain (%)	3 (16.7%)	5 (100%)	**0.002**
Cyclic Upper Abdominal Pain (%)	6 (33.3%)	5 (100%)	**0.01**
Cyclic Chest Pain (%)	3 (60%)	2 (11.1%)	**0.048**
Reoperation (%)	1 (20%)	0 (0%)	0.22
Patient Global Impression of Improvement			
Significant Improvement (%) (Very Much Better and Much Better)	1 (20%)	18 (100%)	**0.001**
Insignificant Improvement (%) (A Little Better and No Change)	4 (80%)	0 (0%)	

Bold *p*-values are the ones that refer to a statistically significant difference between the two groups.

## Data Availability

All data are available from the corresponding author on a reasonable request.
